# Collection of the BD SurePath Pap Test with a broom device plus endocervical brush improves disease detection when compared to the broom device alone or the spatula plus endocervical brush combination

**DOI:** 10.4103/1742-6413.45495

**Published:** 2008-02-12

**Authors:** Sharon Davis-Devine, Sarah J. Day, Amy Anderson, Ashley French, Darcy Madison-Henness, Naomi Mohar, Danielle Tansy, Adarsh Hiremath, Jeffrey A. Douglas, Gregory G. Freund

**Affiliations:** 1 Department of Cytopathology, Carle Clinic, Urbana, IL, USA; 2 Department of Medicine, Carle Clinic, Urbana, IL, USA; 3 Department of Statistics, University of Illinois, Urbana, IL, USA; 4 Department of Pathology, University of Illinois, Urbana, IL, USA

**Keywords:** Collection devices, endocervical cells, liquid-based pap test, surepath pap test

## Abstract

**Objective:**

Here we examine the diagnostic utility of the US Food And Drug Administration (FDA) approved Spatula + endocervical brush combination for the BD SurePath Pap Test (SPPT) and compare it to SPPT collection with the broom alone or to an off-label combination of broom + EC brush. This question is important due to lingering concerns over the value of EC detection to a satisfactory Pap test.

**Methods:**

20,125 SPPT vials were examined for the collection devices contained. The SPPT collection device combinations allowed were: Rovers Cervex-Brush (broom, FDA approved), Medscand Pap Perfect Spatula + Medscand CytoBrush Plus GT (spatula + GT brush, FDA approved) or Rovers Cervex-Brush + Surgipath C-E Brush (broom + CE brush, off label).

**Results:**

Examination of SPPT vials revealed 11,130 collected with the broom, 4,687 collected with the spatula + GT brush and 2,921 collected with the broom + CE brush. Absence of an endocervical/transformation zone was seen in 22.86% of broom cases, 13.10% of spatula + GT brush cases (p= 0.00005 vs broom) and 10.17% of broom + CE brush cases (p= 0.00005 vs broom, p= 0.00005 vs spatula + GT brush). Importantly, LSIL detection was: broom 2.99%; spatula + GT brush 2.45% (p= 0.053 vs broom); broom + CE brush 4.18% (p= 0.034 vs broom, p= 0.0001 vs spatula + GT brush).

**Conclusion:**

When broom + brush combination is compared to broom alone or to spatula + GT brush, the broom + CE brush combination better sampled the endocervical/transformation zone and increased LSIL detection.

## INTRODUCTION

With the issuing of updated recommendations for Pap tests without endocervical cell (EC)/transformational zone (TZ) components, the relevance of EC/TZ presence/absence in routine Pap tests is still pesky.[1] In a previous small study of 37 women, we identified a potential but not significant improvement in EC recovery when multiple Pap collection devices were used to sample the cervix during the BD SurePath Pap Test (SPPT).[2] The purpose of this current study was to determine the efficacy of two commonly used FDA approved SPPT collection device combinations as related to EC recovery and disease detection and to determine if an off-label combination favored by a subgroup of clinicians was acceptable practice. In past work, we have reported that between 3% and 6%[3] of women sampled with the Rovers Cervex-Brush (broom) lacked EC cells. Other groups reporting on broom-type devices have described a range of EC absence from as low as 4.38% to as high as 29.2%.[4–10] Most previous studies, however, focusing on broom-type devices have compared their effectiveness in traditional Pap preparations, and, as we have shown, the SPPT, by itself, reduces limited bys due to lack of ECs by 33% when compared to traditional Paps.[2]

Debate over cervical sampling devices often focuses on EC sampling. Unfortunately, few studies, in which multiple collection devices are compared, report both the EC absence and the abnormal cell detection rate. The retrospective study by Boon *et al*,[5] stands out in that it suggests that there is a correlation between lack of EC recovery with the a broom-type device and reduced detection of CIN III. Most other studies have shown equivalence between spatula + EC brush and broom-type devices. In fact, Buntinx *et al*, in a meta-analysis of 29 trials that included 85,000 patients concluded that there was no significant difference between spatula + cotton swab or EC brush, extended tip spatula or broom-type device in recovery of abnormal cells.[11] This analysis did underscore that use of just an EC brush, cotton swab or Ayre spatula alone was ill-advised. Given the array of collection device choices and our previous work we sought to elucidate the impact of device choice on SPPT quality indicators and diagnoses.

## MATERIALS AND METHODS

*Patient/specimen collection*- After study design approval from the Carle Clinic Association Institutional Review Board, All (n = 20,125) BD SPPT vials (BD, Franklin, NJ) sent to the Carle Clinic Cytopathology Laboratory from July, 2004 to August, 2005 were visually inspected upon acquisition for the collection devices contained. The Pap collection device combinations allowed to providers in the Carle Clinic health care system were: 1) Rovers Cervex-Brush (broom) ((Rovers Cervex-Brush, Rovers Medical Devices, Oss, The Netherlands), 2) Medscand Pap Perfect Spatula + Medscand CytoBrush Plus GT (spatula + GT brush) (CopperSurgical, Trumbull, CT) or 3) Rovers Cervex-Brush + Surgipath C-E Brush (broom + CE brush) (Surgipath, Richmond, IL). SPPTs that did not include one of the approved collection device combinations were excluded from the study. *Cytology-* Diagnostic terminology and quality indicators were derived from the 2001 revision of the Bethesda System (TBS).[12–14] Slides were reviewed following standard practice as we have previously described.[3] All cytology was reviewed blinded to the study.

### Statistics

*P*-values are provided for the null hypotheses that the different sampling methods have the same frequency of quality indicators and disease for each category. Analysis assumes that patients are sampled from identical populations and makes no assumptions about which sampling method is correct. Calculations were performed without use of third-party software.

## RESULTS

Examination of SPPT vials revealed [Table 1] that 11,130 (55.3%) contained the broom alone, 4,687 (23.3%) contained the spatula + GT brush combination and 2,921 (14.55) contained the broom + CE brush combination. Interestingly, 1,387 (6.9%) vials contained none of the approved combinations. The major categories of concern in this subset were no device 79 (0.39%), brush only 166 (0.82%) and spatula only 387 (1.9%). The quality indicator results obtained from the SPPTs via the various qualifying sampling device combinations were as follows [Table 2]: Absence of an endocervical/transformation zone was seen in 22.85% of broom alone cases, 13.10% of spatula + GT brush cases (p= 0.00005 vs broom) and 10.17% of broom + CE brush cases (p= 0.00005 vs broom, p= 0.0001 vs spatula + GT brush). Atrophy was identified in 13.50% of broom alone cases, 10.58% of spatula + GT brush cases (p= 0.00005 vs broom) and 9.35% of broom + CE brush cases (p= 0.00005 vs broom, p= 0.078 vs spatula + GT brush). Unsatisfactory (Unsat) results were obtained in 0.82% of broom alone cases, 2.03% of spatula + GT brush cases (p= 0.00005 vs broom) and 1.64% of broom + CE brush cases (p= 0.00096 vs broom, p= 0.22 vs spatula + GT brush).

**Table 1:** Sampling device combinations observed

	*Device*			
	
	*Broom*	*Spatula+Brush*	*Broom+Brush*	*Other*
Number (%)	11,130 (55.3)	4,687 (23.3)	2,921 (14.5)	1,387 (6.9)

**Table 2:** Observed quality indicators

*Quality indicator*	*Device*			
		
	*Broom (%)*	*Spatula+Brush (%)*	*Broom+Brush (%)*	*Significance p=*
ECs present	6993 (62.83)	3482 (74.29)	2303 (78.84)	BvSB 0.00005
				BvBB 0.00005
				SBvBB 0.00005
ECs absent	2544 (22.85)	614 (13.10)	297 (10.17)	BvSB 0.00005
				BvBB 0.00005
				SBvBB 0.00001
Atrophy	1502 (13.50)	496 (10.58)	273 (9.35)	BvSB 0.00005
				BvBB 0.00005
				SBvBB 0.078
Unsat	91 (0.82)	95 (2.03)	48 (1.64)	BvSB 0.00005
				BvBB 0.00096
				SBvBB 0.22

The diagnoses obtained from the SPPTs via the various qualifying sampling device combinations were as follows [Table 3]. A diagnosis of negative for intraepithelial lesion or malignancy (negative) was rendered in 88.79% of broom cases, 89.29% of spatula + GT brush cases (p= 0.35 vs broom) and 85.31% of broom + CE brush cases (p= 0.00005 vs broom, p= 0.00005 vs spatula + GT brush). A diagnosis of atypical squamous cells of uncertain significance (ASC-US) was rendered in 6.31% of broom cases, 5.57% of spatula + GT brush cases (p= 0.069 vs broom) and 7.84% of broom + CE brush cases (p= 0.0052 vs broom, p= 0.0002 vs spatula + GT brush). A diagnosis of low-grade squamous intraepithelial lesion (LSIL) was rendered in 2.99% of broom cases, 2.45% of spatula + GT brush cases (p=0.053 vs broom) and 4.18% of broom + CE brush cases (p= 0.0034 vs broom, p= 0.0001 vs spatula + GT brush). A diagnosis of high-grade squamous intraepithelial lesion (HSIL) was rendered in 0.27% of broom cases, 0.15% of spatula + GT brush cases (p=0.11 vs broom) and 0.17% of broom + CE brush cases (p= 0.28 vs broom, p= 0.82 vs spatula + GT brush).

**Table 3:** Observed diagnoses

*Diagnosis*	*Device*			
		
	*Broom (%)*	*Spatula+Brush (%)*	*Broom+Brush (%)*	*Significance p=*
Negative	9882 (88.79)	4185 (89.29)	2492 (85.31)	BvSB 0.35
				BvBB 0.00005
				SBvBB 0.00005
ASC-US	702 (6.31)	261 (5.57)	229 (7.84)	BvSB 0.069
				BvBB 0.0052
				SBvBB 0.0002
ASC-H	28 (0.25)	7 (0.15)	7 (0.24)	BvSB 0.17
				BvBB 0.91
				SBvBB 0.40
AGUS	63 (0.57)	15 (0.32)	18 (0.62)	BvSB 0.24
				BvBB 0.76
				SBvBB 0.076
LSIL	333 (2.99)	115 (2.45)	122 (4.18)	BvSB 0.053
				BvBB 0.0034
				SBvBB 0.0001
HSIL	30 (0.27)	7 (0.15)	5 (0.17)	BvSB 0.11
				BvBB 0.28
				SBvBB 0.82
Unsat	1 (0.001)	2 (0.04)	0 (0.0)	Insufficient events for analysis

## DISCUSSION

We have previously shown, in a very small study (n=37), that the all-in-one broom device was essentially equivalent to using a separate spatula and brush in combination.[2] This previous study, however, was limited in power due to its small n. In our current study, we identify significant differences in quality indicators and in diagnoses. Importantly, these differences are sampling device dependent. Table 2 demonstrates that when the broom is compared to the spatula + brush combination EC sampling is much improved with the spatula + brush combination. This finding is notable because, while both device choices are FDA approved, they do not appear equivalent in their ability to sample ECs and the relevance of EC detection to Pap test quality remains somewhat controversial.[15] Recently, Zhao *et al*, found that the presence of EC/TZ significantly improved detection of LSIL and HSIL.[16] Our results do not clearly support those of Zhao *et al.* Table 2 shows that there is a significant increase in EC recovery when the device combinations are used. However, only the broom + brush combination resulted in a significant increase in disease detection and this was only for LSIL (although our study is likely underpowered for interpretation of HSIL and cancer results). A limitation in comparing our work with that of Zhao *et al*, is that Zhao *et al*. investigated ThinPrep Paps and did not report on the sampling devices used. Overall, our data suggests that more than just EC recovery is important for increased disease detection because increased disease detection associated with increased EC recovery was only identified in the broom + brush group. Finally, updated guidelines have been recommended by Davey *et al*, for Paps which lack EC/TZ with a preferred follow-up for a negative being a repeat Pap test in 12 months.[1] Our data support this claim because our increase in disease detection was limited to LSIL and stratified to a collection device combination that is not likely in common usage.

An additional interesting result is the observed difference in the atrophy and unsatisfactory categories when comparing the broom to the spatula + brush combination [Table 2]. The spatula + brush detected significantly less atrophy and had a higher unsatisfactory rate. The atrophy result suggests that the populations sampled by the broom when compared to the spatula + brush combination were not equivalent. The broom + brush quality indicator data in Table 2 argues against this possibility in that atrophy detection and unsatisfactory rate were equivalent to the spatula + brush combination. Therefore, these findings denote that the use of an EC brush device (or use of two devices) enhances recovery of ECs and reduces cases identified as atrophic. Enhanced recovery of ECs by addition of a brush is not unexpected in that improved mechanical travel into the cervical os is anticipated with a narrow diameter device capable of reaching the lower uterine segment. In addition, when compared to the broom, the EC brushes used in this study had bristles that were perpendicular to the handle. The broom device has elongated center "blades" that are designed to extend into the external os. Visualizing the insertion of these "blades" can be difficult due to obstruction by the backbone of the broom where the "blades" attach. Likewise, reduction in atrophy was not entirely unexpected due to better sampling of the endocervical canal. In general, the lateral vaginal wall demonstrates the most consistent morphologic changes related to hormonal status.[17] The face and internal cervix are hormone responsive but not to the same extent as the vagina.[17] Furthermore, conditions of the cervix like prolapse can lead to heightened maturation.[18]

Unanticipated, was the difference in the unsatisfactory rate. Since the sampling techniques using two devices improved EC recovery it was expected that “better” sampling had occurred and cases deemed unsatisfactory would be reduced. Our data show that improved EC recovery is not linked to unsatisfactory rate and supports the rationale for measuring these two quality indicators separately. It was expected that use of two devices would enhance sample collection and lead to a decrease in unsatisfactory rate. Our results do not support this contention and, in fact, point in the opposite direction that multiple devices increase unsatisfactory rate. While the difference between the broom alone and the spatula + brush results are potentially explainable based on the significant design differences between the devices, why the broom + brush differs from the broom alone in terms of unsatisfactory rate is not clear. One possibility is the order of device usage. Our data would indicate that to minimize unsatisfactory rate in the broom + brush setting that the broom be used prior to the brush.

A device-dependent difference in diagnoses was also identified. Interestingly, this difference was only seen with the off-label broom + brush combination and not with the spatula + brush. Table 3 demonstrates that the broom was equivalent to the spatula + brush combination. This finding was not unexpected because comparability to the broom would have been required for subsequent FDA approval for the spatula + brush combination. In comparison the broom + brush device was associated with a significant increases in ASC-US and LSIL diagnoses. Given our findings in Table 2, this result was not entirely unexpected because the broom + brush combination was coupled to a 16% increase in EC recovery indicating improved sampling. Interestingly, the spatula + brush combination was also coupled to increased EC detection (+ 11%) but this combination did not augment ASC-US or LSIL pickup. As noted above, it is not entirely clear why the broom + brush combination out performs the spatula + brush combination. It is likely that the broom's more flexible blades better conform to the ectocervix and, like a toothbrush, has a better ability to retrieve material from a cervix that has lost is smoothness or is irregular in shape. The spreading of the broom blades with pressure also facilitates covering a greater surface area when compared to the fixed reach of the spatula and likely ensures more robust sampling. Sampling of the proximal os is, also, likely facilitated by the broom due to its insertional requirement. In addition, eroded areas, especially at the os, if coupled to channels or creases, are probably better sampled with a flexible device as opposed to a rigid blade. Finally, it was surprising how many cases (6.9%) were sent to our lab without the prescribed device combinations [Table 1]. Of concern were the nearly 2% of cases with spatula only. While a subset of these cases were vaginal and/or cuff preparations, no studies using liquid-based Paps has investigated whether use of just a spatula is adequate. In general, however, use of just a spatula in cervical cytology is not recommended.[5] Outside of hormonal status evaluation of the lateral vaginal wall, it would seem prudent to use an approved or validated device or device combination. In addition, when a SPPT is received by the laboratory, we would recommend examination of the container for the contained sampling devices. This aspect of the SPPT is in contrast to the ThinPrep Pap test where no devices are present in the container and there is no ability to confirm what if any sampling devices were used.

## COMPETING INTEREST STATEMENT BY ALL AUTHORS

No competing interest to declare by any of the authors.

## AUTHORSHIP STATEMENT BY ALL AUTHORS

Each author acknowledges that this final version was read and approved.

According to International Committee of Medical Journal Editors (ICMJE http://www.icmje.org) “author” is generally considered to be someone who has made substantive intellectual contributions to a published study.

Authorship credit should be based on 1) substantial contributions to conception and design, acquisition of data, or analysis and interpretation of data; 2) drafting the article or revising it critically for important intellectual content; and 3) final approval of the version to be published. Authors should meet conditions 1, 2, and 3. Other contributors, who do not meet these criteria for authorship, are listed in an acknowledgments section.

All authors of this article declare that we qualify for authorship as defined by ICMJE http://www.icmje.org/#author.

Each author has participated sufficiently in the work and take public responsibility for appropriate portions of the content of this article.
